# Optimization of composite microbial agents containing plant inter-root growth-promoting bacteria and verification using mini Chinese cabbage (*Brassica rapa* L. ssp. *Pekinensis*)

**DOI:** 10.3389/fpls.2025.1563932

**Published:** 2025-05-21

**Authors:** Xiangjun Fan, Wenxia Yao, Taotao Liu, Yan Wang, Yang Lu, Hongyu Yang, Yuxin Zhang

**Affiliations:** ^1^ College of Horticulture, Gansu Agricultural University, Lanzhou, China; ^2^ Gansu Academy of Agricultural Sciences, 1 New Village, Academy of Agricultural Sciences, Lanzhou, China

**Keywords:** plant growth promoting rhizobacteria, microbial complex, promoted plant growth, correlation analysis, microbial fertilizer

## Abstract

Microbial agent research has developed from single strain to multi-strain complex studies, and Plant growth promoting rhizobacteria (PGPR) has emerged as a hot topic in relation to biofertilizers. Soil environmental degradation problems, such as soil compaction and fertility decline in the Hexi region of Gansu, China, are the focus of this study. The enrichment of PGPR resources and the elucidation of the plant growth-promoting effects of the PGPR strains are also investigated. This study isolated 26-PGPR strains from plant inter-root soils with phosphorous solubilizing ability, 15 strains with nitrogen fixing ability, and 7 strains with IAA secretion ability. The identified strains were from the genera *Pantoea*, *Enterobacteriaceae*, *Acidovorax*, *Brucella*, *Ochrobactru*, *Achromobacter*, *Acinetobacte*, and *Alcaligenes*. Six dominant strains were selected based on the identified characteristics to prepare composite microbial agents, which were then optimized using a pot experiment with mini Chinese cabbage (*Brassica rapa* L. ssp. Pekinensis) and the cross-crossing method. The TE (JQ-MY-41+JQ-MY-42+YC-342) treatment significantly promoted plant growth, and chlorophyll a and carotenoids were increased by 23.1% and 21.2% compared with the CK., respectively. With the TB treatment, Pn increased by 60.5%. The Fv/Fm, ETR, and leaf SOD and POD activities were significantly increased with the TE treatment compared with the TB treatment. Endogenous hormones, such as TZR, GA, and ABA, were significantly increased in TB- and TE-treated plants compared with the CK. Furthermore, organic matter and quick-acting potassium were increased by 33.4% and 71.3%, respectively, and alkaline nitrogen and quick-acting phosphorus were both 2.98 times higher with the TE treatment than with the CK. The correlation analysis of the indicators showed that for the composite mini-Chinese cabbage, TE is the optimal formula, and this will help guide future research and development into microbial fertilizers.

## Introduction

The climatic conditions in Central Gansu, China, are unique and include a high altitude, abundant light, and large temperature differences between day and night. The Mini Chinese cabbage (*Brassica rapa* L. ssp. Pekinensis) has become a staple summer vegetable grown on the plateau (Yin et al., 2023). mini Chinese cabbage one of the important agricultural products in Gansu Province, and in 2019, “Ganzhou mini Chinese cabbage” in the Hexi region was designated as a geographical indication product by the Chinese Ministry of Agriculture. mini Chinese cabbage are characterized by a short planting cycle and high economic benefits, with its average mu yield of 5,000 kilograms and mu profit of about 6,000 yuan, making it the main cash crop in the region. In recent years, over-reliance on chemical fertilizers has resulted in soil degradation, soil erosion, and a reduction in organic matter and beneficial microorganisms in farmland soils in Northwest China, thus creating a challenge for the sustainable development of agriculture in this region (Xiao et al., 2022; Gasparyan et al., 2021). Numerous studies have shown that microflora can promote plant growth, improve the soil environment (Nosheen et al., 2021; Kaur et al., 2022), increase yields, enhance plant resistance to disease and drought, reduce and mitigate plant pests and diseases, and produce a variety of active substances to stimulate and regulate crop growth. Currently, there are a wide variety of microbial strains with various autochthonous and associative nitrogen-fixing microorganisms, such as plant-derived root-promoting bacteria (PGPR) (Gong et al., 2024).

PGPR are microorganisms that live in the soil or colonize plants and can dissolve phosphorus, fix nitrogen, produce Indoleacetic acid (IAA), and promote plant growth (Sati et al., 2022; Harkhani and Sharma, 2024). In recent years, some researchers have investigated the rhizosphere-associated bacteria of soybean (Ai et al., 2022), rice (Kobua et al., 2021), maize (Chen et al., 2021), wheat (Kaur et al., 2021), and tomato (Wang et al., 2021). Several species of inter-root microorganisms such as nitrogen-fixing bacteria of the genus *Azotobacter*, alkali-producing bacteria of the genera *Alcaligens*, *Serratia*, and Bacillus, and other species of microorganisms have disease-preventing and life-promoting potential (Xu et al., 2023). Furthermore, the development and application of phosphorus-fixing, nitrogen-fixing, phytohormone-producing, and disease-suppressing probiotic bacteria as new fertilizers in agricultural production has been investigated (Wang et al., 2021; Yu Y. et al., 2023; Zhang et al., 2020). In addition to plant inter-root biotrophic bacteria, there are also a variety of microbial fertilizers, such as nitrogen-fixing bacterial agents, composite bacterial agents, phosphorus solubilizing bacterial agents, and soil conditioners (Persaud et al., 2023).

Composite microbial agents applied to plant soil can enrich a variety of microorganisms and the inter-root microbial population and improve the soil physicochemical structure to improve and repair the soil environment (Lu et al., 2022; Garcia et al., 2021). Therefore, it is particularly important to explore the screening and isolation of inter-root growth-promoting bacteria and their physiological characterization in mini Chinese cabbage. Zilaie et al. (2022) found that composite microbial agents improved the stress growth of white spurge (*Nitraria tangutorum* Bobrov), which led to a significant increase in the physiological characteristics of the plants. Wang, Q et al. (2023) investigated the effects of composite microbial agents on hydroponically grown lettuce and celery, and the results showed that they improved the agronomic traits of the plants and promoted nutrient uptake and photosynthesis in the root system.

Although there are many studies on the growth promotion of different crops by PGPR, there is a large gap in the research on the customized growth promotion effect of onion rhizosphere growth-promoting bacteria on mini Chinese cabbage in the Hexi Corridor of Gansu Province, and most of the research focuses on a single strain or a simple combination of strains. Compared with traditional research, this study is closer to actual needs and provides a precise fertilizer solution for mini Chinese cabbage cultivation. At the same time, this study adopts a multi-index evaluation system, which not only focuses on yield and growth indicators, but also comprehensively evaluates mini Chinese cabbage quality and soil nutrients, providing more comprehensive and rigorous data support.

In this investigation, phosphorous solubilizing, nitrogen fixing, and IAA bacterial strains were screened and isolated from the inter-roots of plants. The effects of the composite bacterial agents on the growth of mini Chinese cabbage were then investigated using a potting test to provide a theoretical basis for the industrial development of vegetables in this region and future research and development of microbial fertilizers.

## Materials and methods

### Experimental material

Mini Chinese cabbage (*Brassica rapa* L. ssp. *Pekinensis*) was used in this study. Soil samples for the trial were obtained in September 2021 using a five-point sampling method from the inter-root soil of onion (red-skinned onion) with consistent growth along the Hexi area in Gansu (100°53′00″~101°29′00″ E. longitude; 37°42′30″~38°21′00″ N. latitude), China. The altitude of the site is 1570 m, there is 3085 h of average annual sunshine hours, the temperature difference between day and night is 13.00–16.07°C, the average annual temperature is 7°C, the frost-free period is 153 d, the average soil capacity from 0 to 200 cm is 1.376 g·cm^-3^, the organic matter is 17.9 g·kg^-1^, the quick-acting nitrogen is 128.8 mg.kg^-1^, the quick-acting phosphorus is 24.7 mg·kg^-1^, the quick-acting potassium is 82.0 mg·kg^-1^, and the soil pH value is 7.0–8.0, which is slightly alkaline. The study used Luria-Bertani Broth (For bacterial activation), Potato Dextrose Agar (For fungal activation), Skim Milk medium (Semi-fixed nitrogen fixation), King’s medium (For isolation of IAA-producing strains), and Monkina organophosphate media (For isolation of phosphorus solubilizing bacteria).

### Test methods

#### Isolation, screening, purification, and preservation of plant inter-root promoting bacterial strains

Sterile water (99 mL) was added to a conical bottle containing 1 g of rhizosphere soil and vortexed for 1–2 min. It was then placed in a constant temperature shaking table (37°C, 180 rpm, 1 h) for shock culture. The prepared soil suspension absorbed 1 mL of the supernatant and was prepared into diluents with concentrations of 10^-2^, 10^-3^, 10^-4^, and 10–^5^ mmol/L using a dilution gradient method. The prepared dilution solutions for each concentration were inoculated into NFM, NBRIP, and Monjinna organophosphorus media and cultured in an incubator at 28°C for 6 days. Single colonies with faster growth and larger morphologies were selected from the NFM medium for repeated lineation, separation, and purification, i.e. the nitrogen-fixing strain. Single bacterial colonies of different sizes with transparent rings (phosphorus-dissolved rings) around them were selected from the NBRIP and Monjinna organophosphorus media using an inoculation ring. Repeated operations were performed using a plate scribing method until the strains were purified, and they were then stored in the refrigerator at 4°C (Wang X. et al., 2023).

#### Determination of nitrogen fixation, phosphorus solubilization, and IAA secretion activity

A molybdenum antimony colorimetric method was used to determine the phosphorus content in the culture solution, and the calculation formula was as follows (Equation 1):


(1) $$\text{phosphorus content }\left(\mu {\text{g mL}}^{-1}\right)=\left(\text{P}\times \text{V}\times \text{Ts}\right)/\text{V}0$$

(Amri et al., 2023)

An acetylene reduction method was used to determine the nitrogenase activity, and a gas chromatograph (Agilent Technologies GC-7890F) was used to determine CH_4_ production. The chromatographic conditions were as follows: glass column length of 2 m, the inner diameter was 0.4 cm, the stretcher was GDX-502, the oven temperature was 180°C, and a hydrogen flame ionization detector (FID) was used at 170°C. Nitrogen fixation enzyme activity was expressed as nmol C_2_H_4_ mL^-1^ bacterial solution (Amri et al., 2023), and the formula used was as follows (Equation 2):


(2) $$\text{ARA}={\text{h}}_{x}\times \text{C}\times \text{V}/{\text{h}}_{s}\times 24.9\times \text{t}$$

Where hx is the peak value of the sample; hs is the standard concentration of C_2_H_2_ (nmol mL^-1^); V is the volume of the incubation vessel (mL); t is the incubation time of the sample (h); and ARA is the concentration of the produced C_2_H_4_ (nmol mL^-1^ h^-1^).

The IAA concentration (μg mL^-1^) was determined using the S2 colorimetric method (Aasfar et al., 2021).

#### Identification of superior PGPR strains

The DNA of the good probiotic strains was extracted and amplified with bacterial 16S rDNA universal primers (PA: 5′-AGAGTTTGATCCTGGCTCAG-3′; PB: 5′-AAGGAGGTGATCCAGCCGCA-3′) using a DNA extraction kit (OMEGA QN0886) according to the manufacturer’s instructions. The amplified products were examined and sent for sequencing by Lianchuan Biologicals Corporation. The sequences were retrieved from the GenBank database and aligned using Clustal X software and analyzed using Blast software. The phylogenetic tree was constructed using the neighbor-joining method in MEGA 5.0 software (Admassie et al., 2023; Li et al., 2022).

### Experimental design

#### Strain antagonism test

Six strains with high levels of phosphorus solubilization, nitrogen fixation, and IAA production were by plant interaction test. After activation on LB plates, plant interaction test was performed in pairs The test three strains to perform plate test in pairs as a combination, After the six strains were arranged and combined, there were a total of 20 different combinations. Finally the method of determining whether there was antagonism between the strains on the plate was based on whether there was a break between the cross lines.

#### Optimization of composite microbial agent formulations

The three strains on the non-antagonistic plate were mixed into LB liquid medium as a composite microbial agent formulation, with three replicates for each combination. After incubation in a shaking bed at 30°C and 150 rpm for 36–48 h, the bacterial solution was centrifuged (10 min, 12000 rpm) and dilute to 1mL bacterial suspension with an absorbance value of 600.Sequentially, three replicates were taken from each formulation, and 1 mL of bacterial suspension was added to the characteristic culture solution at the same time. The amount of dissolved phosphorus, nitrogen fixation, and IAA production in the composite microbial formulations were then determined (Kumari et al., 2015). Finally, the optimal formulation of the composite microbial agent was screened using Topsis (Technique for Order Preference by Similarity to a ldeal Solution) comprehensive analysis with DPS 15.10 software.

#### Growth promotion test

The three individual strains of the screened optimal formulation were mixed in equal proportions, and 1% surfactant (Compounds that significantly reduce the surface or interfacial tension between two states), 5% protectant (Protection, activation and enhancement of environmental adaptation of microbial strains), and 1% antioxidant (Improved antioxidant capacity of the strain to protect cells from oxidative stress damage) were added (Yuan W. et al., 2022). The pot test was used to determine the growth-promoting effects on Mini Chinese cabbage. The experiment was conducted at the Agricultural Science Building, Gansu Agricultural University, China.

There were five treatments: control CK and TB (GT-51+JQ-MY-41+JQ-271), TC (JQ-MY-41+JQ-232+YC-342), TE (JQ-MY-41+JQ-MY-42+YC-342), and TF (JQ-271+YC-342+JQ-232) as the composite microbial agent formulation. The potting method was used to determine the growth-promoting effect on Mini Chinese cabbage (*Brassica rapa* L. ssp. *Pekinensis*). The Mini Chinese cabbage seedlings were transplanted into pots (10 × 10 cm) containing sterilized substrate: vermiculite at a 5:1 ratio and placed in an incubation room that was 26 ± 1°C with 14 h per day light for cultivation. The experiment was begun on 21 June 2023 in the culture room of the College of Horticulture, Gansu Agricultural University. When the seedlings exhibited 3–4 true leaves, The other treatments were injected with 20 mL of composite microflora to the Mini Chinese cabbage seedlings. and the control CK was injected with 20 mL of fermentation solution without bacteria. The bacterial solution was watered 10 times throughout the process at 5 d intervals, and index measurements were performed in mid-August.

### Indicator measurement

#### Determination of morphological indicators

After the 30-day treatment (end of July), plant height, stem diameter, and plant dry matter were determined using a tape measure, vernier calipers, and an electronic balance, respectively (Zhou et al., 2021; Zhang et al., 2024; Inada et al., 2007).

#### Determination of chlorophyll content and fluorescence parameters

Chlorophyll content was determined using the method described by Xie et al. Chlorophyll fluorescence parameters in mini cabbage leaves were measured using a Maxi Imaging PAM Chlorophyll Fluorometer, and initial fluorescence (Fo), maximum fluorescence (Fm), steady-state fluorescence (Fs), maximum fluorescence yield (Fm0), and initial fluorescence in the light (Fo0) were determined in sequence. Finally, Fv/Fm, Y(II), qP, qN, and the electron transfer rate were calculated for PSII (1-qP) (Tomaszewska-Sowa et al., 2022; Ma et al., 2023). The equations used for these calculations were as follows (Equations 3–7):


(3) Fv/Fm =(Fm-Fo)/Fm

(4) $$\text{ΦPSII}=\left(\text{F}{\text{m}}^{\prime }-\text{Fs}\right)/\text{F}{\text{m}}^{\prime }$$

(5) $$\text{qP}=\left(\text{F}{\text{m}}^{\prime }-\text{Fs}\right)/\left(\text{F}{\text{m}}^{\prime }-\text{F}{\text{o}}^{\prime }\right)$$

(6) qN=1−(Fm′−Fo′)/(Fm−Fo)

(7) ETR=ΦPSII×0.5×0.84

#### Determination of photosynthetic gas exchange parameters

After the 30-day treatment (end of July), photosynthetic gas exchange parameters (Gs, Pn, Ci, and Tr) were measured using a Ciras-2 portable photosynthesizer (PP Systems, Amesbury, MA, USA) (Zheng et al., 2023).

#### Endogenous hormone measurement

High performance liquid chromatography (HPLC) was used to determine the levels of six endogenous hormones, namely, ZT (zeatin), tZT (anit zeaxanthin), IAA (auxin), ABA (abscisic acid), SA (salicylic acid), and GA_3_ (gibberellin), the leaves of Mini Chinese cabbage. The HPLC conditions were as follows: HPLC model Waters e2695, ZORBAX SB-C18 column (4.6 × 150 mm, 5 µm), and the mobile phases of methanol (45%), water (50%), and acetic acid (5%) mixed solution. The mobile phase was a mixture of methanol (45%), water (50%), and acetic acid (5%) with isocratic elution at a flow rate of 1 mL min^-1^, an injection volume of 10 µL, a detection wavelength of 254 nm, and a column temperature of 30°C (Inada et al., 2007).

#### Antioxidant enzyme activity assay

The mini Chinese cabbage fresh leaf samples (0.5 g) were homogenized with a pestle and mortar in an ice-cold mortar containing 6 mL of ice-cold 50 mM sodium phosphate buffer (pH 7.0), which contained 0.2 mM EDTA and 1% (w/v) polyvinylpyrrolidone. The homogenate was filtered through gauze, and the supernatant (enzyme extract) was collected at 15,000 × *g* to determine the superoxide dismutase (SOD) (Yang et al., 2024), catalase (CAT) (Liu X. et al., 2023), and peroxidase (POD) (Sun et al., 2023) activity Jun et al. Three measurements were obtained for each sample.

#### Determination of soil physical and chemical properties conclusions

Quick-acting phosphorus was determined using a molybdenum antimony colorimetric method (Yang Y. et al., 2023). Quick-acting potassium was measured using an ammonium acetate-flame photometric method (Ma et al., 2022). The alkaline nitrogen was measured using an alkaline dissolution diffusion method (Chen et al., 2023). Organic matter was measured using the dichromate dilution heat method (Wang Z. et al., 2023). The total nitrogen content was determined using a Tecator1030 fully automatic nitrogen determinator (Niu et al., 2022). Total phosphorus was determined using an M410 flame photometer (Yang L. et al., 2023). Whole potassium was determined using a CARY50 UV-visible spectrophotometry (Perveen et al., 2023).

#### Statistical analysis

The experimental results were compiled and analyzed using WPS Office Excel, SPSS 22.0 software, principal component analysis (PCA) using factor analysis, statistical analysis using one-way ANOVA of LSD to compare the significant level of differences (p< 0.05), correlation analysis using Pearson’s correlation coefficient method. Graphs were plotted using Origin2022.

#### Experimental flowchart

**Figure 1:**
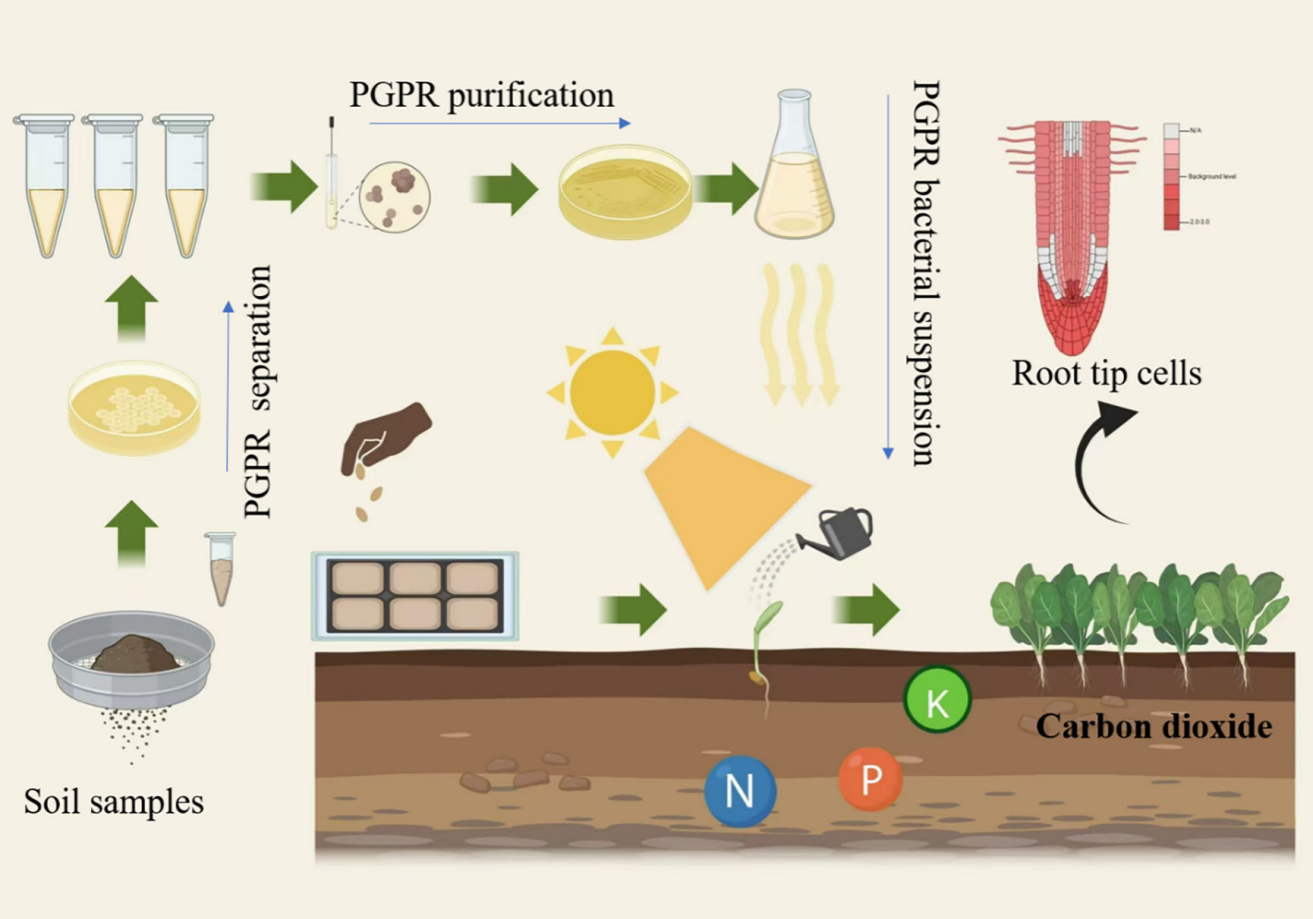
Flowchart of the promoting effect of plant rhizosphere promoting bacteria compound microbial agent on the growth of *mini chinese cabbage*.

The experimental flowchart is shown in **Figure 1**.

## Results

### Isolation and identification of strains

Twenty-six functional strains were screened, all of which could dissolve phosphorous, 15 could fix nitrogen, and 7 could secrete IAA. Combined with the phosphorous solubilization and nitrogen fixation of each strain, six dominant strains (**Table 1**) were selected for the potting test: JQ-MY-41, JQ-MY-42, JQ-271, YC-342, JQ-232(4), and GT-151. Phosphorous solubilization and nitrogen fixation of JQ-MY-42 were the highest at 455.833 μg mL^-1^ and 90.021 nmol.h^-1^ mL^-1^, respectively. The pH values of all strains were acidic and significantly different.

**Table 1:** Phosphorus solubilization, nitrogen fixation and bacterial inhibition by six dominant strains of bacteria.

Different strains	quantity of dissolved phosphorus	Amount of phosphorus dissolved	PH value	Bacteriostatic rate
JQ-MY-41	75.55 ± 0.511 e	14.15 ± 0.240 d	4.43 ± 0.012 e	0.12± 0.008 b
JQ-MY-42	318.59 ± 0.982 c	90.44 ± 0.455 a	5.45 ± 0.020 b	0.12 ± 0.027 b
JQ-271	419.28 ± 0.972 b	7.03 ± 0.051 e	4.30 ± 0.005 f	0.13 ± 0.005 b
JQ-232(4)	77.67 ± 0.464 e	49.72 ± 0.484 b	4.82 ± 0.012 d	0.08 ± 0.014 b
YC-342	447.70 ± 0.953 a	4.47 ± 0.234 f	5.61 ± 0.014 a	0.19 ± 0.019 a
GT-151	161.54 ± 0.524 d	23.91 ± 0.504 c	5.04 ± 0.023 c	0.10 ± 0.008 b

Different lowercase letters indicate significant differences between treatments (P < 0.05).

**Figure 2:**
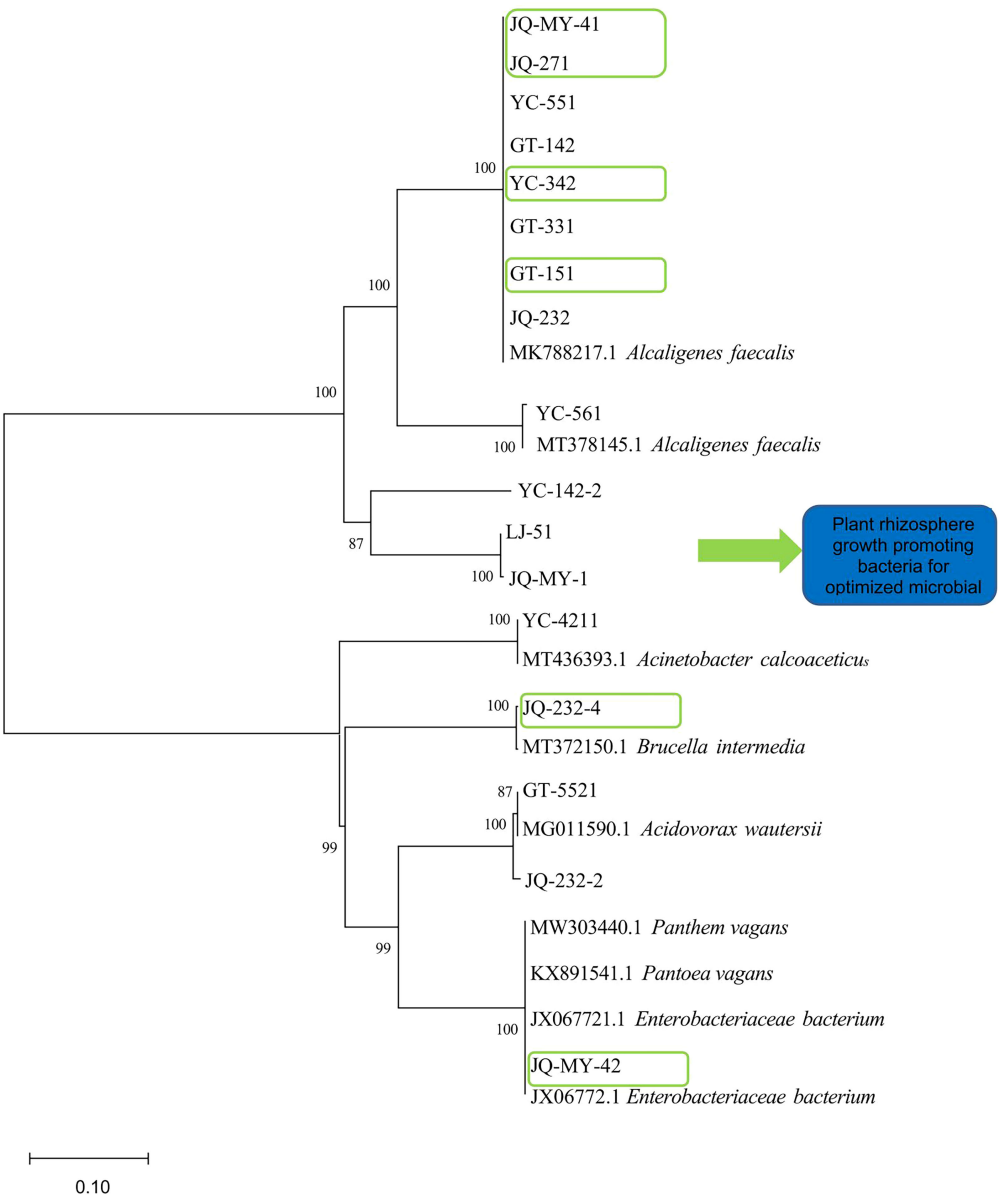
Construction of a phylogenetic tree for onion inter-root promoting bacteria based on 16S r DNA sequences (Note: Phylogenetic tree preceded by Gen Bank accession number; Scale is the nucleotide substitution rate; A total of eight genera are shown, namely *Pantoea, Enterobacteriaceae, Acidovorax, Brucella, Ochrobactrum, Achromobacter, Acinetobacte*, and *Alcaligenes*).

The sequencing results for the strains were compared with homologous sequences belonging to *Pantoea*, *Enterobacteriaceae*, *Acidovorax*, *Brucella*, *Ochrobactrum*, *Achromobacter*, *Acinetobacter*, and *Alcaligenes*, and a phylogenetic tree was constructed as shown in (**Figure 2**).

### Optimization of the composite microbial agent formulation

**Figure 3:**
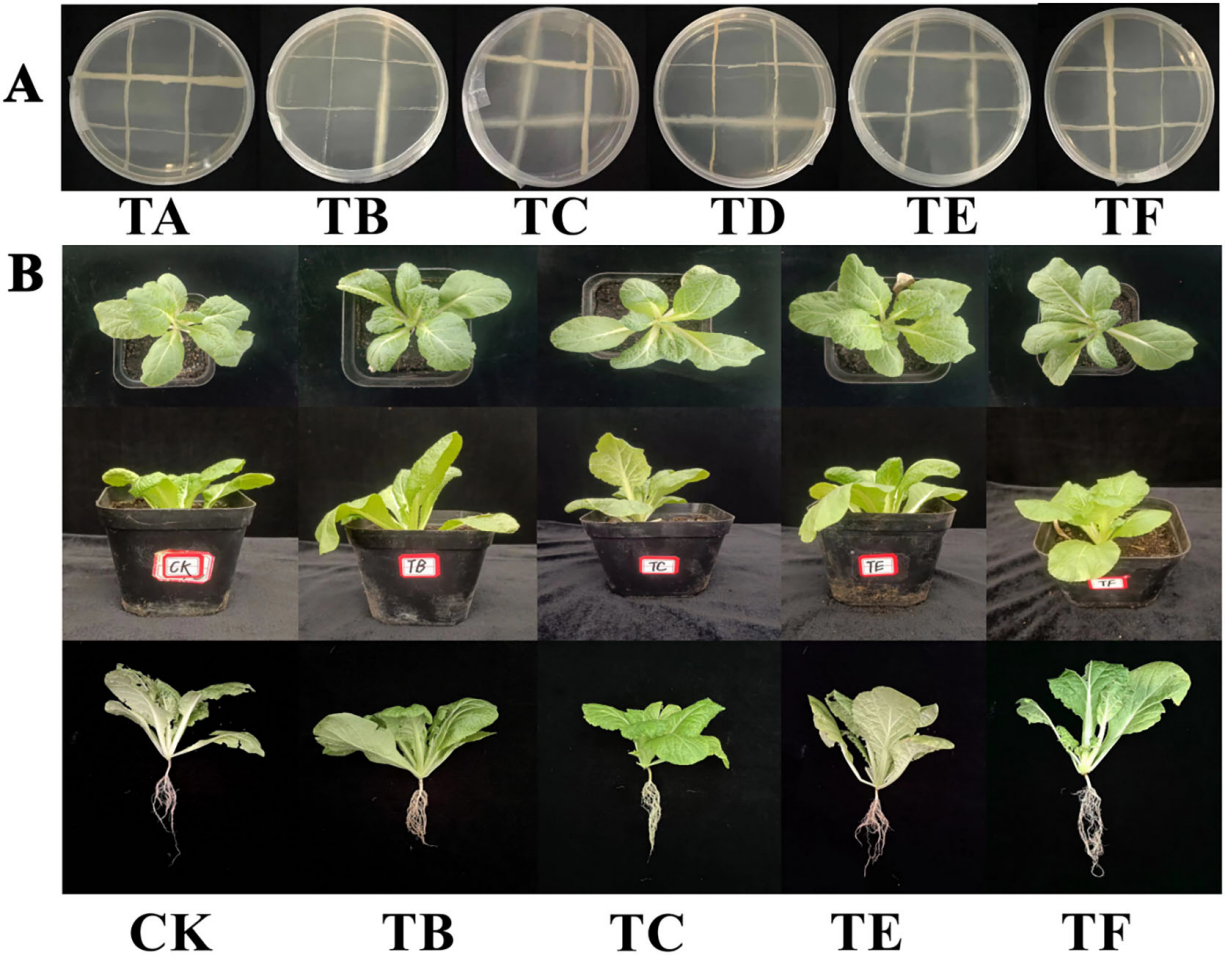
Antagonism between different combinations and the growth pattern of mini Chinese cabbage plants after treatment [Note: **(A)**: antagonistic effect between strains; **(B)**: growth pattern of mini Chinese cabbage plants under different treatments. TA(JQ-232+ JQ-MY-42+ JQ-271); TB(GT-151+JQ-MY-41+JQ-271); TC(JQ-MY-41+JQ-232+ YC-342); TD(JQ-MY-41+ JQ-232+ JQ-MY-42); TE(JQ-MY-41+JQ-MY-42+YC-342); TF(JQ-271+ JQ-232+ YC-342)].

There was no disconnection in the two-by-two crosses of each strain, indicating that there was no antagonism (**Figure 3A**). The phosphorous solubilization, nitrogen fixation, and IAA production capacities of the strain combinations after compounding are shown in **Table 2**. Except for TA and TD, the phosphorous solubilization effects of the TB, TC, and TF combinations were better and above 300 µg/mL, and the phosphorous solubilization effect of the TE combination was the best at 611.70 µg/mL. The nitrogen fixation capacity of the strain combinations, except for the TD combination, was good, and the secretion of IAA was > 4 mg/L in the TB and TE combinations, which was twice as much as that in the TA and TD combinations. The TOPSIS comprehensive analysis of each good combination, according to the statistics (CI) and ranked from largest to smallest, was as follows: TE > TB > TF > TC > TD > TA. Higher statistical rankings indicate that the combination has a stronger comprehensive ability, and the four optimal composite microbial agent formulations were further used in the pot experiments.

**Table 2:** Comprehensive analysis of the amount of phosphorus solubilization, nitrogen fixation and IAA secretion by composite bacterial agents.

Excellent combination	The amount of phosphorus dissolved	Amount of phosphorus dissolved	IAA	pH value	Topsis analyze
TA	176.91 ± 13.09 c	210.52 ± 3.21 c	1.59 ± 0.07 c	3.34 ± 0.014 e	0.36
TB	439.90 ± 14.01 b	395.69 ± 7.92 a	4.20 ± 0.69 ab	3.81 ± 0.040 c	0.78
TC	399.97 ± 48.76 b	266.28 ± 14.31 bc	3.98 ± 0.71 ab	5.28 ± 0.012 b	0.53
TD	201.29 ± 55.75 c	128.86 ± 10.45 d	2.01 ± 0.19 bc	3.51 ± 0.030 e	0.41
TE	611.70 ± 84.99 a	311.15 ± 41.40 b	4.31 ± 0.99 a	3.78 ± 0.015 a	0.82
TF	439.79 ± 24.98 b	393.22 ± 28.55 a	3.59 ± 0.76 abc	6.21 ± 0.017 a	0.58

Different lowercase letters indicate significant differences between treatments (P < 0.05).

#### Effect of complex microbial agents on plant morphology in the growth of mini Chinese cabbage

The different combinations of composite microbial agents had significant effects on the morphology of the mini Chinese cabbage plants (**Figure 3B**). The morphology of the untreated plants is shown with the CK, and the plants had reduced leaf areas, number, and root systems. The TB, TC, TE, and TF were the optimal combinations of the composite microbial agents plant morphology under treatment.

#### Changes in growth and photosynthetic pigment content of mini Chinese cabbage with complex microbial agents

**Figure 4:**
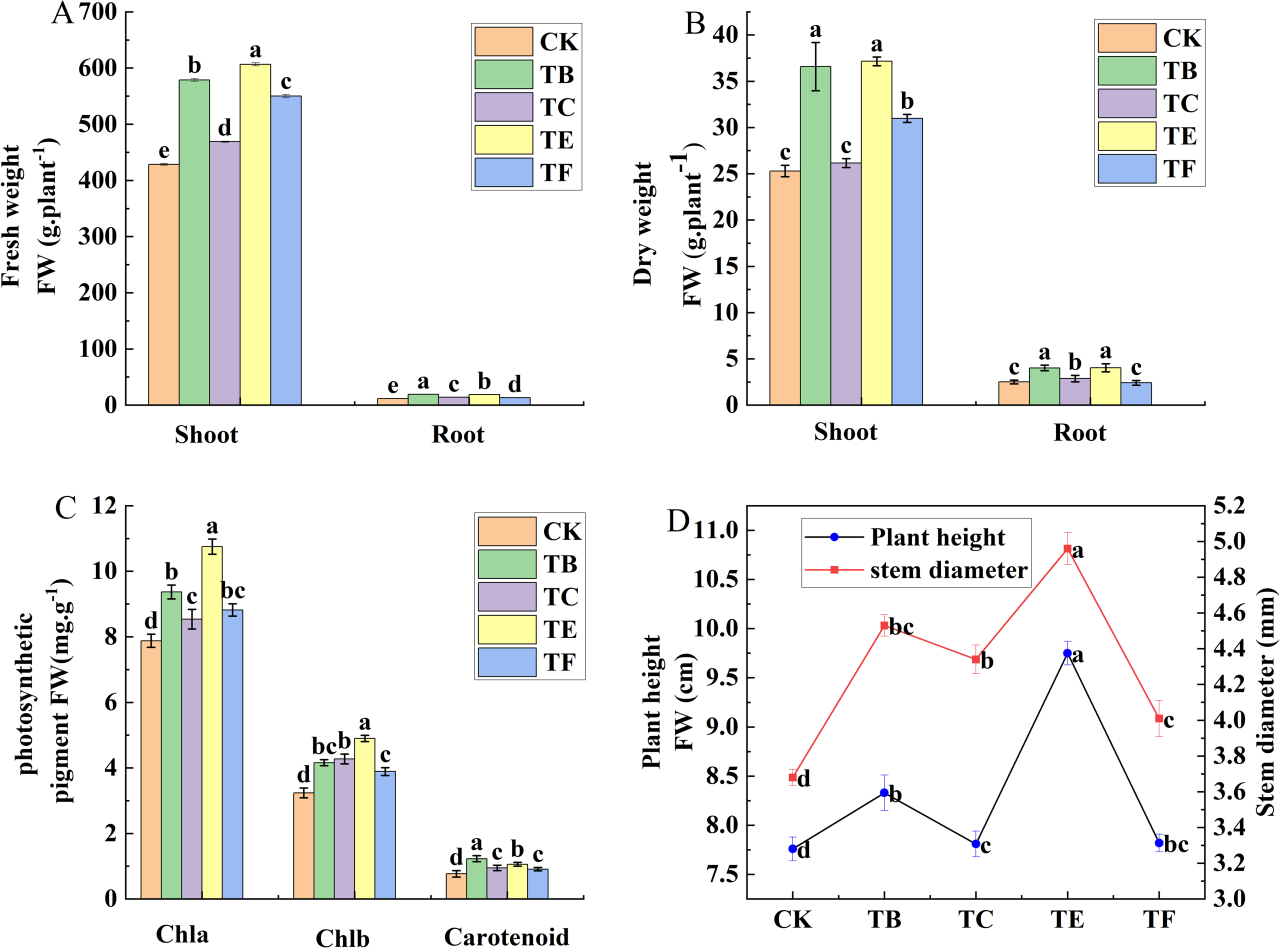
Effect of composite microbial agents on the growth and photosynthetic pigment content of mini Chinese cabbage [Note: **(A)** fresh weight; **(B)** fresh weight; **(C)** chlorophyll content; **(D)** plant height stem thickness]. Different lowercase letters indicate significant differences between treatments (P < 0.05).

The effects of different composite microbial agent treatments on the growth of mini Chinese cabbage are shown in (**Figure 4**). Compared with the CK, the composite microbial agent TE treatment significantly increased plant height, stem thickness, and above-ground and below-ground dry and fresh weight. The TC treatment resulted in a relatively small increase in plant height 10.9%, stem thickness increased by 19.1%, above-ground dry weight was 1.43, above-ground fresh weight 1.39 times that of CK treatment, and belowground dry weight increased by 34.1%, above-round fresh weight 17.7%. Except for TE, the TB treatment showed a greater increase compared with CK, with 21.4%, 22.9%, 30.6%, and 35.8% increases in plant height, stem thickness, and above-ground and below-ground dry matter mass, respectively (**Figures 4A-C**). Compared to CK, TB treatment increased chlorophyll a by 33.1% and carotenoids by 32.1%, and TE treatment increased chlorophyll a by 23.1% and carotenoids by 21.2%. The chlorophyll b content decreased in all treatments compared with that in the CK, and was reduced by 13.1% with the TE treatment (**Figure 4D**).

#### Effect of complex microbial agents on the photosynthetic parameters of mini Chinese cabbage leaves

**Figure 5:**
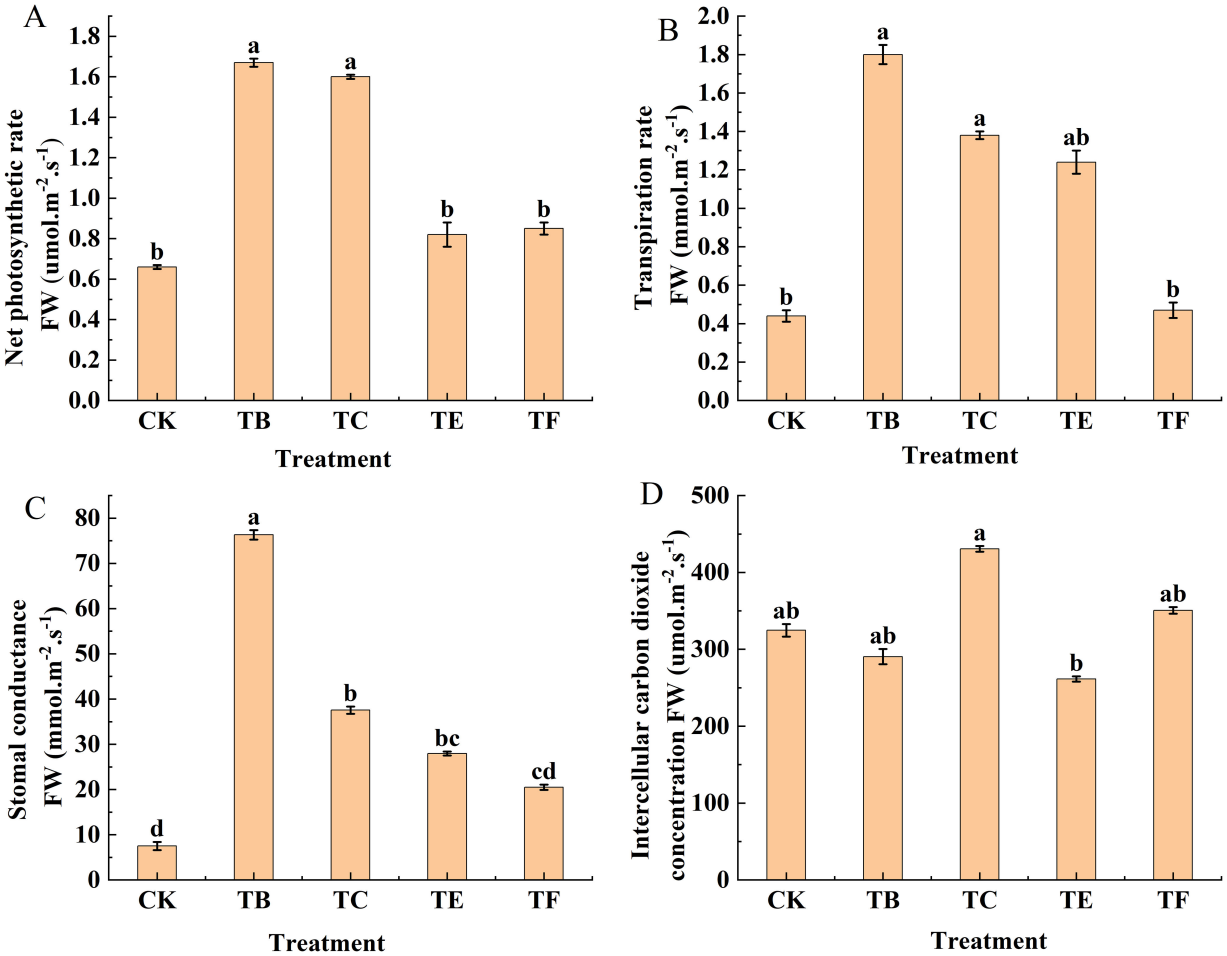
Effect of composite bacterial agents on light and gas exchange parameters of mini Chinese cabbage [Note: **(A)** net photosynthetic rate (Pn); **(B)** transpiration rate (Tr); **(C)** stomatal conductance (Gs); **(D)** intercellular carbon dioxide concentration (Ci)]. Different lowercase letters indicate significant differences between treatments (P < 0.05).

The photosynthetic parameters of mini Chinese cabbage leaves under different treatments are shown in (**Figure 5**). The net photosynthetic rate (Pn) of the onion inter-root composite microbial agent on mini Chinese cabbage leaves varied significantly among the treatments. The index value of the TB treatment was largest and differed significantly from that of the TE and TF treatments, and the TC treatment was more statistically significant, but all four were significantly higher than that of the CK treatment and increased by 60.5%, 19.5%, 21.1%, and 55.6%, respectively. The difference between transpiration rates (Tr) with the TF treatment and CK was not statistically significant, but both were significantly smaller than those with TB, TC, and TE treatments. The largest transpiration rate was with the TB treatment, which was 4.09 and 3.82 times higher than that of the CK and TF treatments, respectively. Compared to CK, stomatal conductance (Gs) increased most statistically significant with the application of different mycorrhizal microbial agents with 90.1% increase in TB treatment, 79.9% increase in TC, 73.1% increase in TE, and 63.3% increase in TF. The intercellular CO_2_ concentration (Ci) was greatest with the TC treatment, and it differed significantly from the CK, but the CK was significantly higher than the TB and TE treatments. The TF treatment did not show a significant difference compared with the CK and increased by 7.37%. Overall, the TB treatment was the most effective.

#### Effect of complex microbial agents on the fluorescence parameters of mini Chinese cabbage

**Figure 6:**
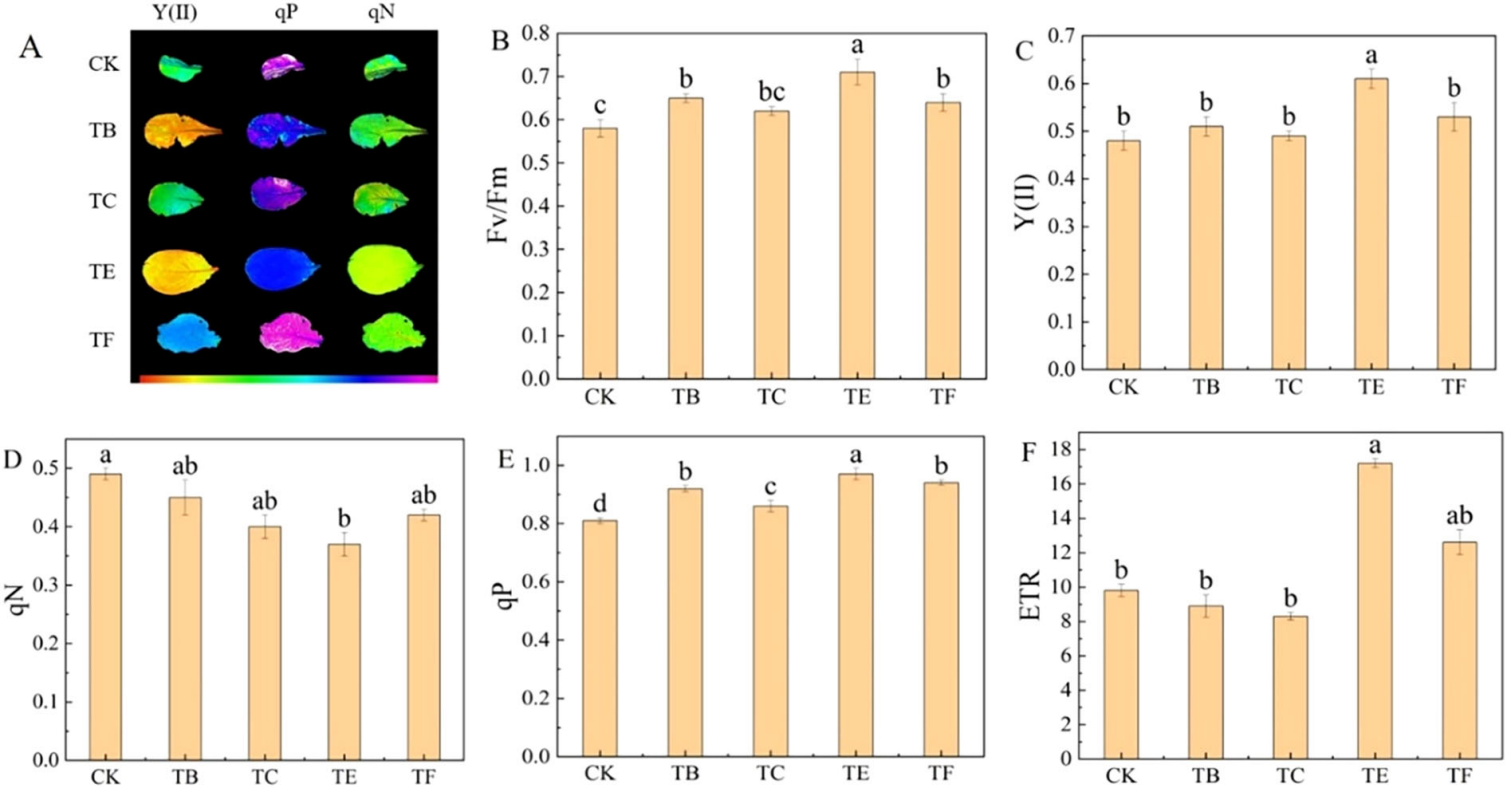
Effect of composite microbial agents on chlorophyll fluorescence parameters of mini Chinese cabbage [Note: **(A)** images of *Y(II)*, qP, and *qN*; **(B)** maximum efficiency of *PSII* photochemistry (*Fv/Fm*); **(C)** actual photochemical efficiency of PSII, [*Y(II)*]; **(D)** non-photochemical quenching coefficient (*qN*); **(E)** photochemical quenching coefficient (*qP*); **(F)** relative electron transfer rate *(ETR*)]. Different lowercase letters indicate significant differences between treatments (P < 0.05).

The chlorophyll fluorescence of the mini Chinese cabbage from different composite fungal treatments is shown in (**Figure 6**). The TC treatment significantly reduced the actual photochemical efficiency (Y(II)), non-photochemical burst coefficient (qN), and relative electron transfer rate (ETR) of PSII compared with the CK. The maximum photochemical efficiency (Fv/Fm) of PS II was reduced by 17.8%, qN by 20.5%, and ETR by 44.6% in TE treatment compared with CK In addition, the TE treatment significantly increased the photochemical burst coefficient (qP) compared with the CK, but there was no significant difference with the TC treatment (**Figure 6E**).

#### Effect of complex microbial agents on the enzyme activity of mini Chinese cabbage leaves

**Figure 7:**
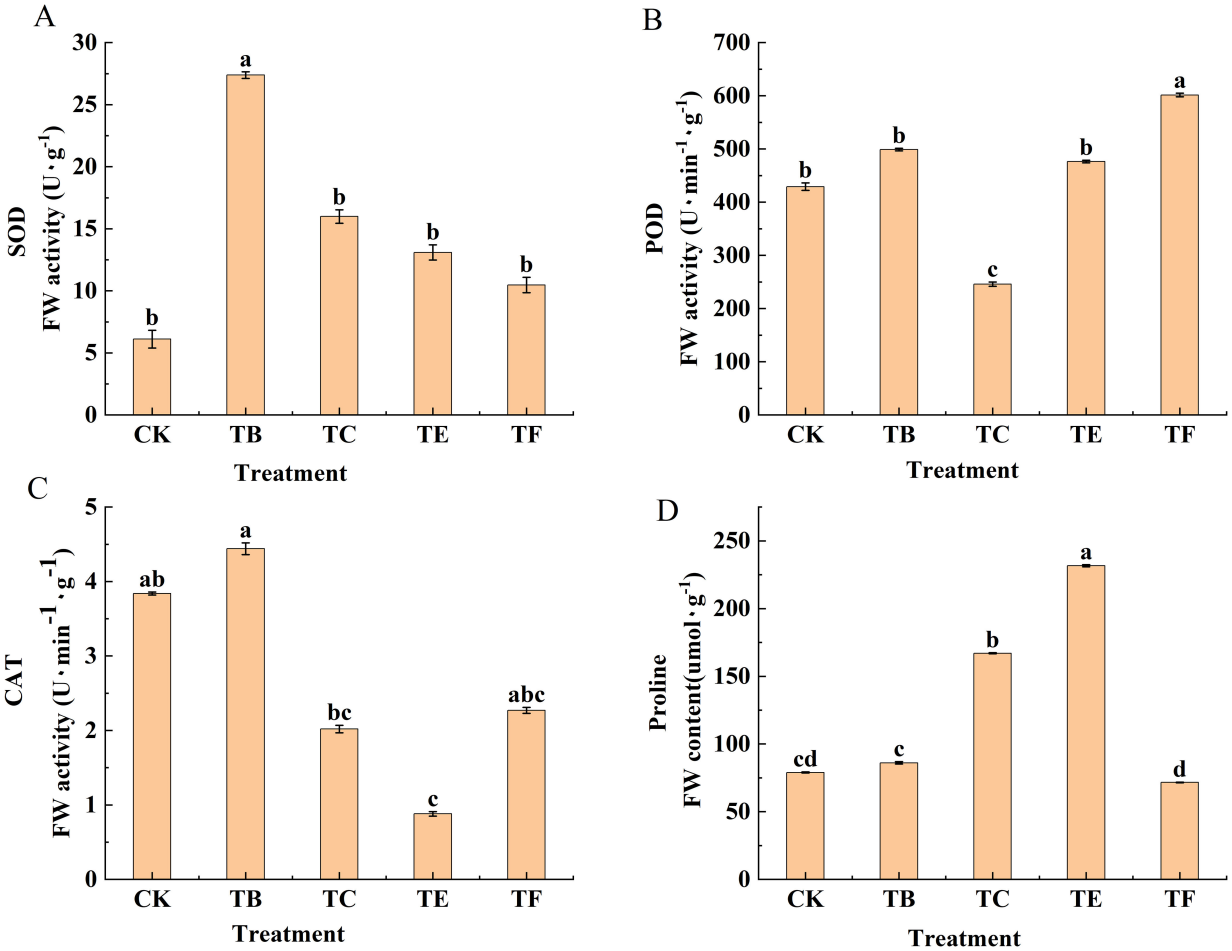
Effect of composite bacterial agents on antioxidant enzyme activities of mini Chinese cabbage [Note: **(A)** superoxide dismutase (SOD); **(B)** peroxidase (POD); **(C)** catalase (CAT); **(D)** proline (Pro)]. Different lowercase letters indicate significant differences between treatments (P < 0.05).

The enzyme activity of the mini Chinese cabbage leaves from the different treatments is shown in (**Figure 7**). The SOD activity of the mini Chinese cabbage leaves shows a trend of first increasing and then decreasing, and the TB treatment SOD enzyme activity peaked at 25.13 U g^-1^, which is 5 times higher than that of the CK. The TF treatment showed a smaller increase compared with the other treatments, which increased by 37.26% compared with the CK. The POD activity was the same in TB- and TE-treated mini Chinese cabbage leaves. The POD activity when watering with the bacterial solution was significantly higher than that of the CK, and the highest POD activity level was 651.32 U g^-1^ with the TF treatment, and this was 33.6% higher than that of the CK. Compared to CK, the POD activity of TB and TE treatments increased by 1.21 and 1.19 times, respectively, while that of TC treatment decreased by 39.5%. The differences in the CAT content in the mini Chinese cabbage leaves with the different treatments was significant. Except for the TB treatment, the TC, TE, and TF treatments decreased by 47%, 64%, and 28%, respectively, compared with CK. The TB treatment was significantly increased by 17% when compared with the CK, and was 1.91, 4.63, and 1.67 times greater when compared with the TC, TE, and TF. Proline content when watering with composite microbial agent was significantly increased by 54.3% and 69.5% with the TE and TC treatments, respectively, compared with CK. The TB and TF treatments had smaller increases of 20.2% and 7.71%, respectively when compared with the CK. The proline content of the TE treatment was increased by 68% compared with that of the TF treatment, and there was a significant difference between the treatments.

#### Effect of compound microbial agents on the hormone content of mini Chinese cabbage leaves

**Figure 8:**
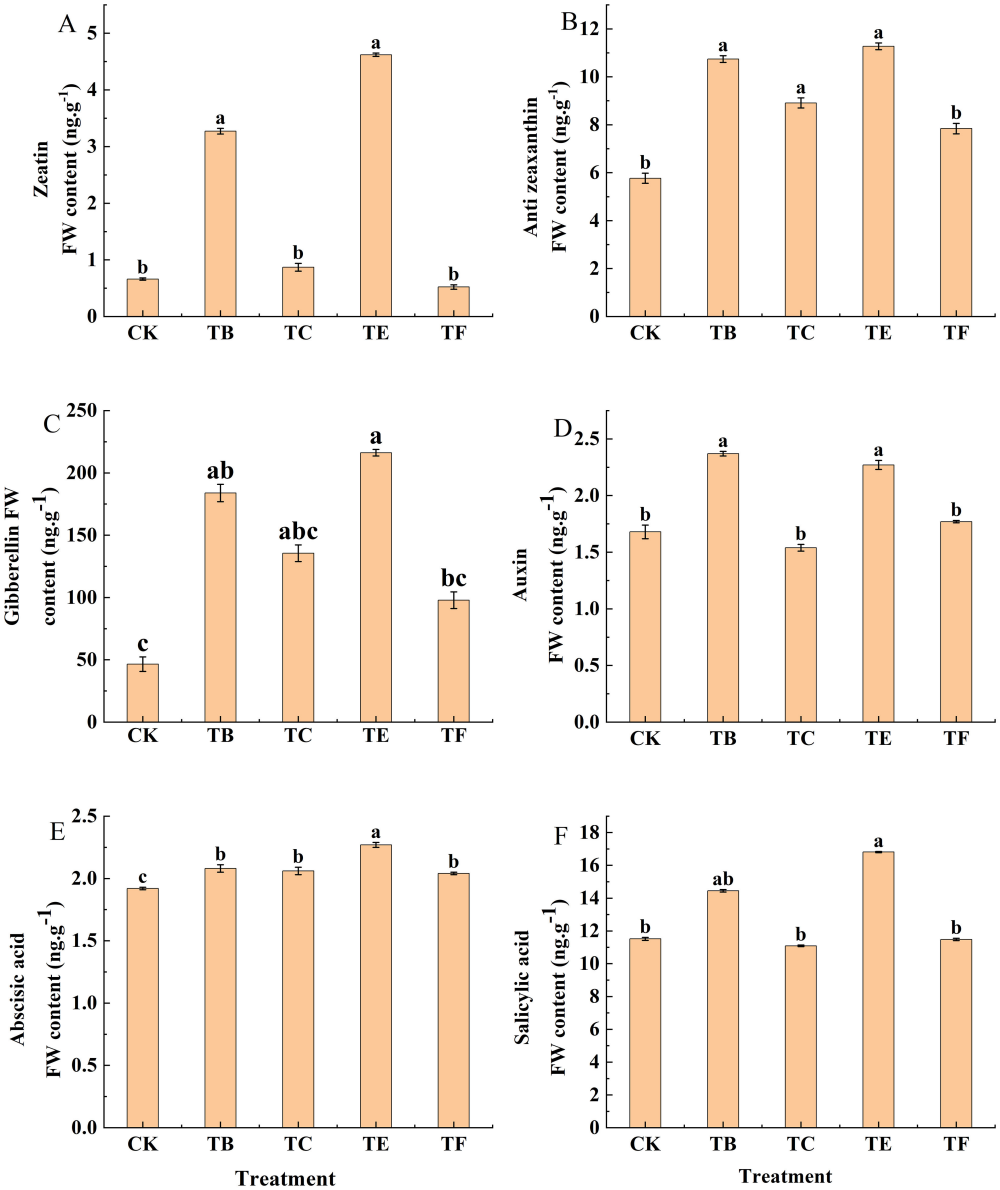
Effect of composite microbial agents on endogenous hormone content of mini Chinese cabbage leaves [Note: **(A)** zeatin (ZT); **(B)** trans-zeatin (TZR); **(C)** gibberellin (GA); **(D)** growth hormone (IAA); **(E)** abscisic acid (ABA); **(F)** salicylic acid (SA), the results are expressed as the mean of three replicates ± SE, and the different letters denote the significant differences between the treatments (P<0.05)]. Different lowercase letters indicate significant differences between treatments (P < 0.05).

Changes in the leaf endogenous hormone contents are shown in (**Figure 8**). The ZT content in the leaf was significantly different between the treatments (*p*< 0.05), in which the TB and TE treatments significantly increased by 75.6% and 82.1% compared with the CK. The TC treatment was less significant difference compared with the CK, and it increased by 13.5%, and the TF treatment was decreased from CK at 0.62 ng g^-1^. The endogenous hormones TZR and GA were optimized by the TB treatment, which increased TZR by 46.2% and GA by 47.7% in the TB treatment compared to CK, while TZR increased by 70.2% and GA by 77.6% in the TE treatment; however, the differences between the two treatments, TE and TB, were not significant. The IAA content in the leaves increased after the application of the composite microbial agent, Compared with CK, IAA increased by 34.1% in the TB treatment; it decreased in the TC treatment and increased in all other treatments. The ABA content increased in all treatments when compared with the CK, and the differences between the treatments were more significant with the TB, TC, TE, and TF, which were 1.17, 1.15, 1.38 and 1.13 times that of the CK. Salicylic acid (SA) was significantly increased in the TB and TE treatments (19.1% and 30.2%, respectively) when compared with the CK. The differences between the TC and TF treatments were not significant compared with the CK.

#### Effect of complex microbial agents on the physico-chemical properties of mini Chinese cabbage soil

**Figure 9:**
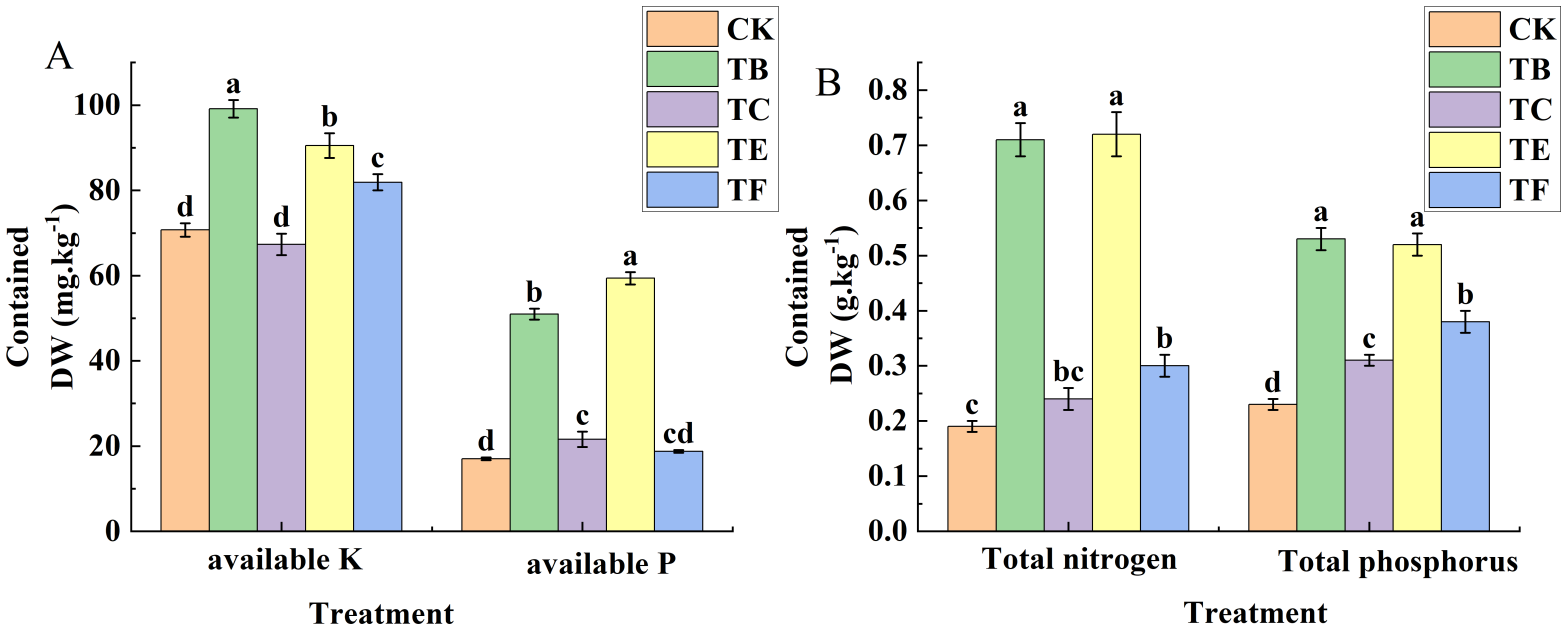
Effect of composite microbial agents on physico-chemical properties of soil for growing mini Chinese cabbage [Note: **(A)** quick-acting potassium (K), quick-acting potassium (P); **(B)** total nitrogen, total phosphorus)]. Different lowercase letters indicate significant differences between treatments (P < 0.05).

**Figure 10:**
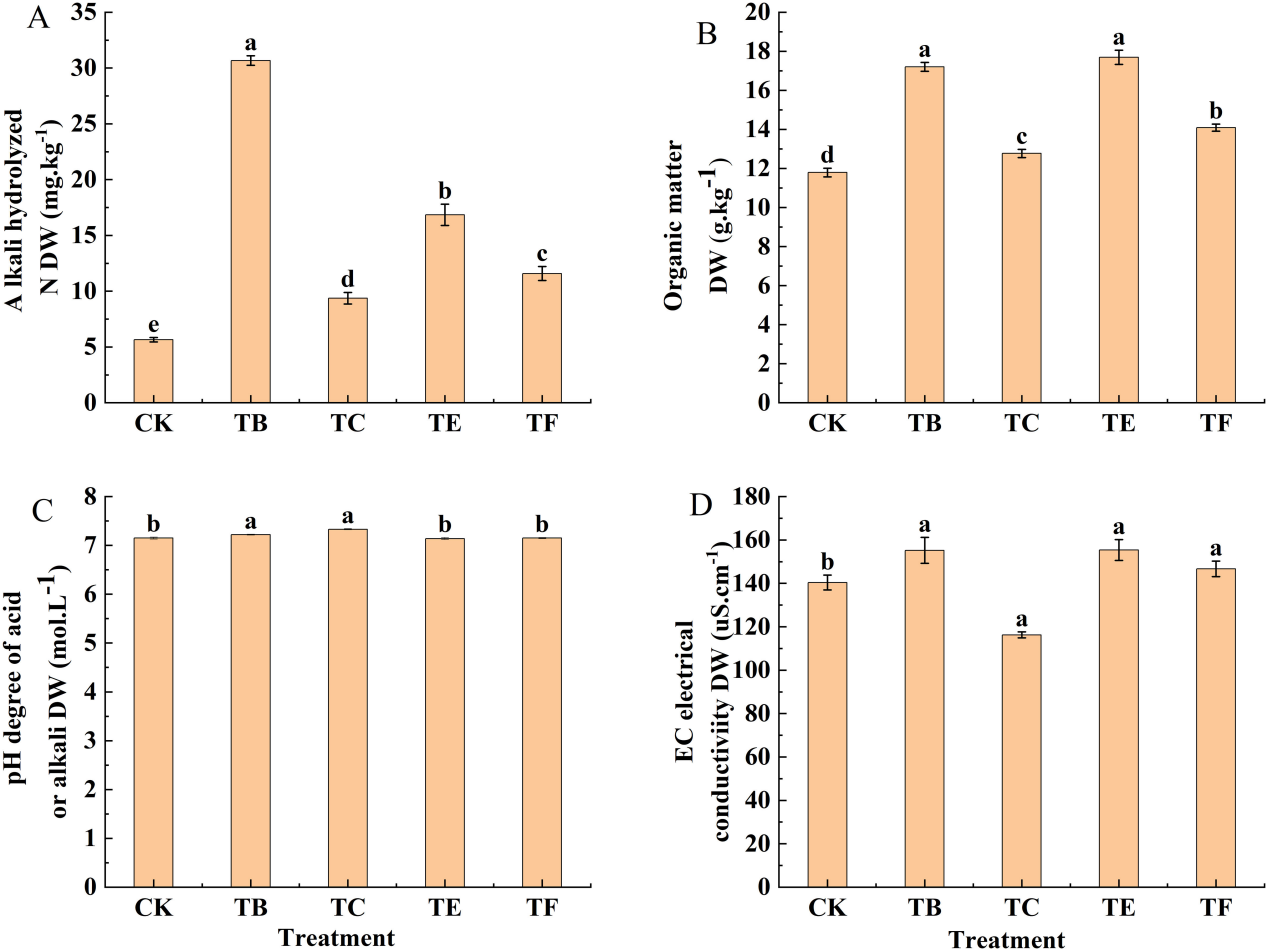
Effect of composite microbial agents on physico-chemical properties of soil for growing mini Chinese cabbage. [Note: **(A)**: alkali hydrolyzed nitrogen; **(B)**: organic matter; **(C)**: PH degree of acid or alkali; **(D)**: electrical conductivity]. Different lowercase letters indicate significant differences between treatments (P < 0.05).

As shown in (**Figure 9**), The rapid available phosphorus analysis showed that the TB and TE treatments were No significant difference, the results showed a 3.6% increase with the TB treatment when compared with the TE, and the optimal results were obtained with the TB treatment, which had a soil quick-acting phosphorus content of 99.14 mg·kg^-1^. For the rapidly available potassium, the TE treatment was significantly higher than the other treatments, with an increase of 71.3% compared with the CK, and TC and TF treatments were No significant differences compared with the CK, with 21.0% and 7.62%, respectively. The effects of the TB and TE treatments in alkaline dissolved nitrogen were significant and 3.48 and 2.98 times that of the CK. The TC and TF treatments also increased significantly compared with the CK and were 39.7% and 51.2%, respectively. Organic matter with quick-acting phosphorus, quick-acting potassium, and alkaline dissolved nitrogen changes in line with the TE treatment content and was significantly higher when compared with the other treatments, and the significant difference was small for the TB and TE organic matter, which increased by 31.4% and 33.4%, when compared with the CK, and the treatment of the TC organic matter was smallest at 12.77 g. kg^-1^(**Figure 10**).

### Comprehensive evaluation of the physiological effects of compound microbial agents on mini Chinese cabbage

#### Correlation analysis of physiological indexes in mini Chinese cabbage

**Figure 11:**
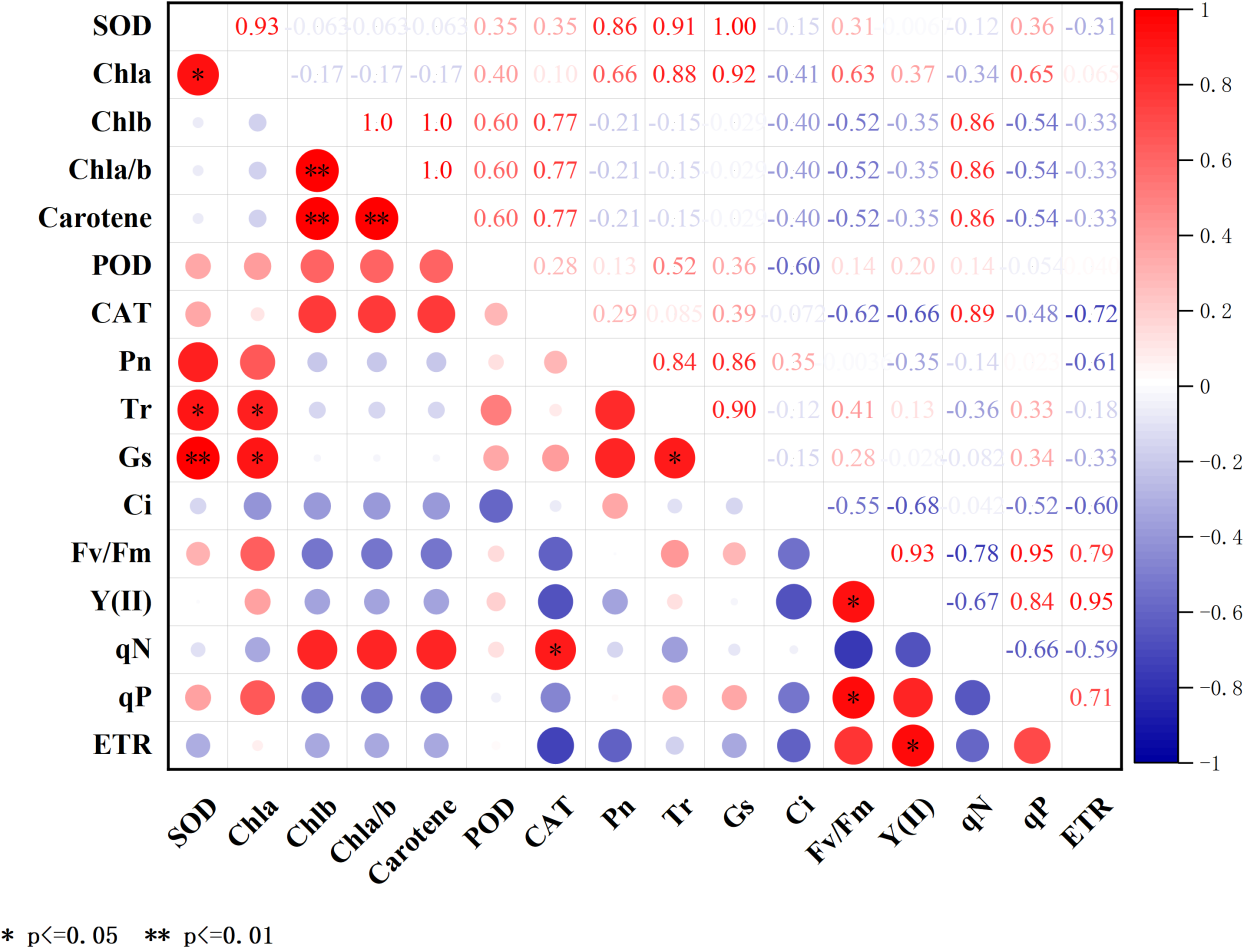
Pearson’s correlation analysis of the effect of composite microbial agent treatments on the physiological characteristics of mini Chinese cabbage [Note: * represents significant correlation (P< 0.05), * * represents significant correlation (P< 0.05)].

The physiological indices of the different composite fungal treatments on the leaves of *C. cattleya* were correlated (**Figure 11**). The results showed that there were several sets of positive correlations between the chlorophyll and chlorophyll fluorescence parameters and antioxidant enzymes. There were significant positive correlations between chlorophyll a and SOD, and Tr and Gs, and the antioxidant enzyme SOD was significantly correlated with the transpiration rate (Tr) and significantly correlated with stomatal conductance Gs, respectively. In addition, chlorophyll b was highly significantly positively correlated with carotenoids and the electron transfer rate ETR was significantly correlated with the actual photochemical efficiency Y(II) of PSII in the leaves of mini Chinese cabbage.

#### Principal component analysis of physiological indexes of mini Chinese cabbage by different treatments of composite microbial agents

**Figure 12:**
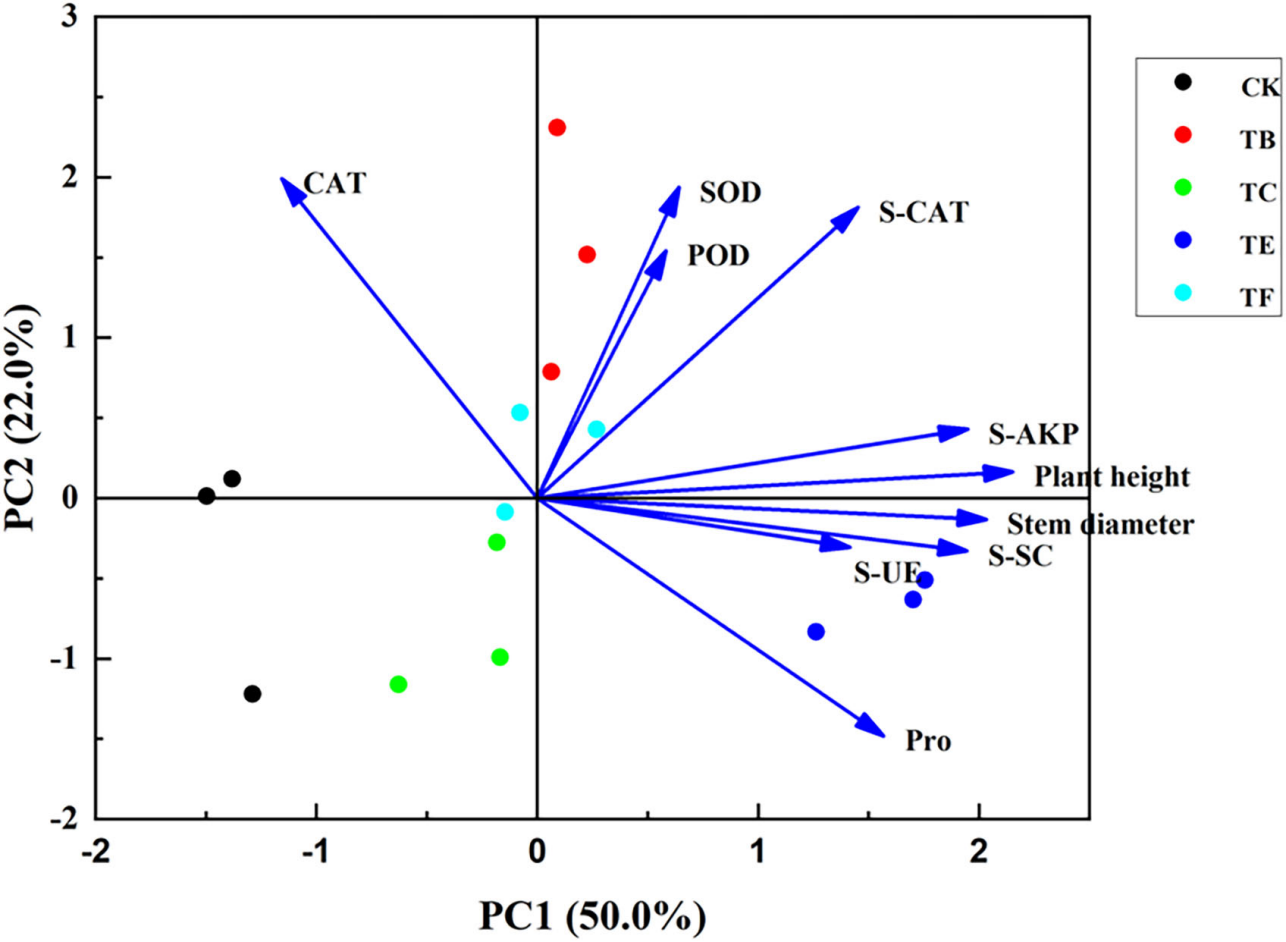
Principal component analysis of physiological indexes of mini Chinese cabbage after compound microbial agents treatment.

The principal component analysis (PCA) was performed on the baby vegetables under different composite microbial agents formulations. As can be seen from (**Figure 12**), the principal component analysis (PCA) of plant height, stem thickness, leaf enzyme activity, and soil enzyme activity showed that there were three principal components greater than the eigenvalue of 1. The cumulative variance contribution ratio of PC1 and PC2 reached 72.0%, which indicated that these two principal components were able to reflect 72.0% of the information of these 10 indexes. Except for CAT, all other indicators were positively correlated with PC1; plant height, SOD, POD, and S-AKP were positively correlated with PC2, and stem thickness, Pro, S-SC, and S-UE were negatively correlated with PC2, with TE and TB treatments having a significant effect on each indicator.

The composite score (F) was the sum of the product for each principal component analysis and the relative variance contribution ratio, i.e., F = F1 × 41.623% + F2 × 31.498% + F2 × 21.944%. The combined scores for the mini Chinese cabbage under different treatments were (CK), −0.7399 (GT-51+JQ-MY-41+JQ-271), −0.7652 (JQ-MY-41+JQ-232+YC-342), 0.77264 (JQ-MY-41+JQ-MY-42+YC-342), and 1.81114 (JQ 271+YC-342+JQ-232), as shown in **Table 3**. The ranking of the effects of different concentrations of ABA on the physiological effects of the potato under stress was in the following order: T3 > T4 > T2 > T1 > CK.

**Table 3:** Comprehensive evaluation analysis of composite microbial agents in different treatments.

Treatment	Principal component score			Comprehensive score (F)	Comprehensive Score ranking
	PCI (F1)	PC2 (F2)	PC3 (F3)		
CK	-0.632715	0.200135	0.131001	-0.301579	5
TB	-0.092357	0.528797	0.045182	0.481622	2
TC	0.066971	0.013865	-0.324775	-0.243939	4
TE	0.506668	-0.075576	0.242959	0.674051	1
TF	0.151436	-0.266951	-0.094365	-0.209880	3

Composite ranking score: ranking= F1 x variance contribution ratio +…. Fn x variance contribution ratio.

## Discussion

Different types of PGPR have different mechanisms for promoting plant growth. Glick (2014) found that plant growth-promoting bacteria containing ACC deaminase reduce the ACC level in plants. Organisms that produce ACC deaminase reduce the ethylene level in plants, making them more resistant to growth inhibition under various ethylene-induced stresses. Pii et al. (2007) confirmed that Sinorhizobium meliloti can produce NO through enzyme activity similar to NO synthase, and believed that NO may be involved in the induction of nodules in the roots of Medicago truncatula, thereby increasing the nitrogen content of plants. Bacillus amyloliquefaciens FZB42 is considered to be a PGPR that has a significant impact on plant growth. While producing IAA, it can also produce secondary metabolites with antibacterial and antifungal activity, changing the composition of soil microorganisms (Pii et al., 2015).

With the development of modern eco-agriculture and the increasing awareness of sustainable development, composite microbial agent made of PGPR are gradually replacing chemical fertilizers (Chebotar et al., 2022). For example, The application of a synthetic fertilizer containing biotrophic microbial agent by Kobua et al. (2021) resulted in an increase in the maize (*Oryza sativa*) growth rate (PFG), and a 4%-5% increase in thousand grain weight (1000-GW), and a 14% increase in grain yield. Wang Y. et al. (2021) found that PGPR increased the relative abundance of nitrogenous spirochetes and slow-growing rhizobacteria in soil using a pot experiment. In this study, composite microbial agent prepared from PGPR strains that were isolated and screened from the onion inter-root were used to irrigate the roots of mini cabbage seedling. The results showed that they promoted the physiological indexes of the cress plant, including height, stem thickness, and dry matter mass. Specifically, the TE composite microbial agents treatment showed the most significant effects in promoting the growth of mini cabbage seedling. This was similar to the results of Li et al. (2023), who investigated the growth of Moso bamboo and found that different composite bacterial agents can significantly affect the physiological and biochemical indexes of the plant.

Photosynthesis describes the conversion of solar energy into chemical energy in plants, and this series of reaction changes cannot be separated from photosynthetic pigment molecules (Zhou Y. et al., 2023). Chlorophyll is the primary photosynthesis pigment in plants, and the present experimental study showed that the Chla and carotene of mini cabbage seedling leaves were increased significantly after the application of microbial agents compared with the CK. This may be because of the reduced Na^+^ in the mini cabbage seedling leaves, and the chlorophyll content is closely related to the photosynthetic rate (Liu X. et al., 2023). In this study, the chlorophyll content and Pn of the mini cabbage leaves showed an increasing trend with the application of mycorrhizal treatments. Among them, the net photosynthetic rate (Pn) increased by 60.5% at most, which is similar to the results of Wen et al. who treated non-heading Chinese cabbage with a microbial agent prepared by a single strain and found that the net photosynthetic rate of the plant increased significantly by 26.2%. This may be because the metabolites produced by the microorganisms were contained in the soil during fermentation and reproduction, such as endogenous hormones and other plant growth regulators, and this directly promote the growth of mini cabbage seedling, which in turn increases the accumulation of chlorophyll and improves the rate of net photosynthesis. This is similar to the conclusion drawn by Santos and Rigobelo (2021) in their study on sugarcane, where bacterial strains were found to promote plant growth.

According to You et al. (2022), only a simultaneous decrease in Ci with Gs indicates that the decrease in Pn is caused by stomatal limitation; if Gs decreases while Ci remains unchanged or increases, the decrease in Pn is caused by non-stomatal factors such as a decrease in the assimilative capacity of the leaf saprophytes. The results showed that the application of microbial fungi significantly increased Pn and Gs and decreased Ci, indicating that microbial fungi increased the photosynthetic efficiency of *C. napus*, primarily by promoting the opening of leaf stomata, which allowed more CO_2_ to enter into the chloroplasts for assimilation, and this was similar to the results of Sun et al. (2023) for *S. napus*.

Chlorophyll fluorescence is a probe of photosynthesis, and a series of important regulatory processes within the photosynthetic apparatus can be understood by analyzing fluorescence parameters (Ding et al., 2022). Zhou et al. found that PGPR increased the photosynthetic rate of seagrass hosts (Zhou et al., 2024). The inoculation of onion inter-root probiotic bacteria in this experiment had a significant effect on fluorescence parameters such as Fv/Fm, actual photochemical efficiency of PSII Y(II), qP, qN, and ETR of mini cabbage seedling seedlings. The different biotrophic agents play a role in the growth and development of plant seedlings, but their effects on plant light and physiological metabolic parameters varied, possibly because of their different biotrophic mechanisms (Tanveer et al., 2023) The growth promotion results were similar to those of Neshat et al. (2022) for oilseed rape inoculated with PGPR.

Plants senescence during growth and the production of reactive oxygen species (ROS) in their cells that can damage the cell membrane plasma structure, leading to premature senescence, thus affecting the normal developmental cycle. SOD, POD, and CAT are important antioxidant enzymes, and when a plant experiences adversity stress, they scavenge ROS to reduce damage (Zhang et al., 2022). Jiao et al. (2024) showed that PGPR inoculations in apple plants enabled the inter-root complexes of probiotic bacteria to eliminate the effects of accelerated senescence on tomato somatic cells and promote plant growth by increasing SOD activity in the leaves. The results of this study show that the application of different composite bacterial agents in baby vegetables showed an increasing trend in leaf antioxidant enzyme activities compared with the CK. Among them, the SOD and POD contents were 7.26 times and 26.0.7 times respectively, which were compared with the research on promoting the growth of baby cabbage by Wang et al. (2022) using microbial agents that have been put into production This indicates that the application of onion inter-root composite microbial agents can act on plants and increase antioxidant enzyme activity to reduce the accumulation of reactive oxygen species and delay leaf senescence. This may be because soil microorganisms secrete beneficial active substances to optimize the inter-root environment after root irrigation, promote the uptake and utilization of nutrients by the root system, increase the accumulation of minerals and microelements, and activate the activity of antioxidant enzymes in the plant to improve its resistance (Niu et al., 2022). The accumulation of proline (Pro) helps maintain osmoregulation in plant cells and protects macromolecules and cell membrane tissues under water-deficient conditions (Yu X. et al., 2023). The different treatments of the composite in this study induced the accumulation of Pro in the leaves of mini cabbage seedling, which proves that the onion inter-root promoting composite stabilizes the osmotic stress of the plant and promotes growth and development.

Plant endogenous hormones are organic compounds produced in the plant in trace amounts that regulate their own physiological processes (Zheng et al., 2023). Romero-Muñoz et al. (2022) investigated the effects of PGPR on the growth of escarole and showed that both ground cover and AMF inoculations increased the activity of active Gibberellins and cytokinins. The results showed that the inoculation of onion inter-root composite microbial agent increased the content of endogenous plant hormones (IAA, ABA, SA, and GA), and that IAA can regulate root development and structure to increase the uptake of water and nutrients by the root system during plant growth and development (Zhou Q. et al., 2023). In addition, ABA, a stress hormone accumulated in plants, can alleviate the damage of water stress on plants, and the results showed that the significant differences between the composite microbial agent treatments were small compared to the CK, which indicated that microbial agents may not have an obvious response mechanism to plant leaf ABA content, and that the effects of microbial agents on plant leaf ABA should be further investigated as an external stress (Jia et al., 2022).

The application of microbial agents affects the soil nutrient cycling process, which in turn affects the nutrient uptake and growth of vegetative plants. In this study, complex microbial agents significantly increased the soil content of fast-acting phosphorus and fast-acting potassium, possibly because onion inter-root complex microbial agents dissolve phosphorus, solubilize potassium, and fix nitrogen. In a previous study, strains with several growth-promoting effects were screened from the soil, and when added to soil, they significantly increased the quick-acting phosphorus and quick-acting potassium content (Biswas et al., 2022). Wen et al. (2021) also found that two different microbial agents increased the nitrogen content of plants by 15.1% and 22.4%, which is similar to the results of this experiment that microbial agents increased the nitrogen content of plants. In this study, the TC treatment significantly reduced the alkaline nitrogen content of the soil by the microbial fungi when compared with the other treatments, and this may be because the complex microbial fungi promoted the growth and development of plants, and the above-ground part of the plants absorbed part of the alkaline nitrogen, thus reducing the levels in the soil (Yuan F. et al., 2022).

To further analyze the effects of different composite microbial agent treatments on chlorophyll fluorescence, the photosynthetic parameters and antioxidant enzymes in mini cabbage seedling leaves were analyzed using Pearson’s correlation heat map analysis for each index in this experiment, and there was a significant positive correlation between the chlorophyll and photosynthetic parameters Tr, Gs, and the enzyme activity indexes such as SOD (Liu Y et al., 2023). The results indicate that the increase in chlorophyll content after the application of microbial agents increased the photosynthetic and antioxidant enzyme activities of the plants to a certain extent to promote growth. Multiple indicators have been converted into a small number of comprehensive indicators using principal component analysis to provide a comprehensive evaluation of the indicators of mini cabbage seedling under composite microbial agent treatment (Jiang et al., 2022). In this study, the effects of composite microbial agents on plants were significantly higher than those of CK, and the comprehensive ranking results showed that TB treatment was optimal, which is consistent with previous findings.

## Conclusion

In this study, the effects of PGPR and composite microbial formulation optimization in rhizosphere soil on the promotion of mini Chinese cabbage growth were investigated. Strain antagonism experiments, phosphorous solubilization, nitrogenase activity, and IAA production ability were used to assess the composite microbial formulations. This resulted in the identification of an optimal formulation, which was used in subsequent pot experiments. The results showed that the TE (JQ-MY-41+JQ-MY-42+YC-342) treatment had a significant effect on plant growth and had a multi-faceted catalytic effect. It not only improved the quality of plant dry and fresh matter, but also enhanced the activity of leaf antioxidant enzymes, regulated photosynthetic parameters, enriched chlorophyll content, and optimized chlorophyll fluorescence parameters. In addition, it also regulated the endogenous hormone levels in plants and significantly improved the physical and chemical properties of the soil, compared with the microbial agent prepared by a single strain, it has a stronger growth-promoting effect These findings provide fertilizer support for the future development of the mini Chinese cabbage industry, and provide scientific and technological support for the development and utilization of composite microbial agents specifically for mini Chinese cabbage, promoting the development of sustainable green agriculture.

## Data Availability

The original contributions presented in the study are included in the article/Supplementary Material. Further inquiries can be directed to the corresponding authors.
